# Gender differences in esophageal variceal bleeding in the United States

**DOI:** 10.1080/07853890.2022.2104920

**Published:** 2022-08-05

**Authors:** Aalam Sohal, Hunza Chaudhry, Armaan Dhaliwal, Piyush Singla, Gagan Gupta, Raghav Sharma, Dino Dukovic, Devang Prajapati

**Affiliations:** a Department of Internal Medicine, University of California, Fresno, CA, USA; b Department of Internal Medicine, University of Arizona, South Campus-Tucson, AZ, USA; c Dayanand Medical College and Hospital, Punjab, India; d Punjab Institute of Medical Sciences, Punjab, India; e Ross University School of Medicine, Bridgetown, Barbados; f Department of Gastroenterology and Hepatology, University of California, Fresno, CA, USA

**Keywords:** Gender, variceal bleeding, cirrhosis, disparities

## Abstract

**Background and Aims:**

Esophageal variceal bleeding is a common reason for hospitalization in patients with cirrhosis. The main objective of this study was to analyze the effects of gender differences on outcomes in hospitalizations related to Esophageal variceal bleeding in the United States.

**Methods:**

A retrospective observational cohort study was performed using the National Inpatient Sample (NIS) database for all hospitalizations with a discharge diagnosis of esophageal varices with hemorrhage from 2016 to 2019. The primary outcome was in-hospital mortality, while secondary outcomes included rate of early endoscopy (defined as less than 1 day), AKI, blood transfusion, sepsis, ICU admission and TIPS (Transjugular Intrahepatic Portosystemic Shunt). We also compared the length of stay and total hospitalization charges.

**Results:**

We identified a total of 166,760 patients with variceal bleeding of which 32.7% were females. In-hospital mortality was higher in males, 9.91%, compared to females, 8.31% (adjusted odds ratio (aOR): 0.88, p-value=.008, when adjusted for confounding factors). The odds of undergoing an EGD, length of stay, or total hospitalization charges did not differ between the two groups. Compared to men, women had lower odds of receiving TIPS (aOR = 0.83, p-value=.002).

**Conclusion:**

Women hospitalised with esophageal variceal bleeding are at a lower risk of death compared to males. Further research is needed to elucidate the factors associated with this lower risk.

## Introduction

The natural history of liver disease differs by gender [1]. Women are significantly less likely to have chronic liver disease, with men accounting for 55%–70% of the total cases [2–5] and are thought to have a more favourable clinical course. Women are at a lower risk of progression of liver fibrosis from both viral hepatitis and non-alcoholic steatohepatitis (NASH) [6–8]. This difference in fibrosis progression can be due to the protective effects of sex hormones and decreased incidence of cofactors for fibrosis progression [8]. It has also been documented that women have lower rates of hepatic decompensation as compared to men [9].

Acute esophageal variceal bleeding is a direct consequence of portal hypertension and continues to be one of the most lethal complications of cirrhosis. Over the past three decades, mortality due to variceal bleeding has steadily decreased with improved endoscopic and pharmacological treatments [10]. Despite this improvement in treatment modalities, bleeding from esophageal varices continues to have high mortality (15%–20%) in patients with underlying cirrhosis [11].

It has been noted that variceal bleeding outcomes vary by gender in different countries. A study of 266 patients in Norway revealed that women with cirrhosis and variceal bleeding are at a lower risk of death than males [12]. A similar study on an Italian database showed lower in-hospital mortality in females when admitted with variceal bleeding than their counterparts [13]. To the best of our knowledge, no study has evaluated the effect of gender on esophageal variceal bleeding in the United States. Given the limited data on outcomes of patients with variceal bleeding in males versus females, our study aims to assess the differences in mortality, length of stay (LOS), and hospital costs and charges for patients admitted for esophageal variceal bleeding.

## Methodology

### Data source

The National Inpatient Sample (NIS), maintained by the Healthcare Cost and Utilization Project (HCUP) of the Agency for Healthcare Research and Quality, is the largest database of inpatient hospital stays in the United States [14]. The NIS collects data from a 20% stratified sample of United States hospitals from 37 states and has been reliably used to estimate disease burden and outcomes. Each hospitalization is de-identified and maintained in the NIS as a unique entry with one primary discharge diagnosis and up to 39 secondary diagnoses during that hospitalization, depending on the year of data collection. Each entry includes carries patient demographics, including age, sex, race, insurance status, primary and secondary procedures (up to 25), hospitalization outcome, total charges and LOS. IRB approval was not required as it is publicly available de-identified data.

### Study population

The International Classification of Diseases 10th Version, Clinical Modification (ICD-10 CM) diagnosis codes were used to identify patients (≥18 years) hospitalized with esophageal variceal bleeding admitted between 2016 and 2019. All patients admitted electively were excluded due to the inconsistency of elective admission and the emergent nature of acute variceal bleeding. Cases with missing mortality data, gender status, or demographic information were excluded. In total, 166,760 cases met the inclusion criteria.

### Study outcomes and variables

The primary outcome was comparing inpatient mortality from esophageal variceal bleeding between males and females. We also compared the mean LOS, total hospitalization cost and charges between males and females as a surrogate marker for healthcare cost utilization. Hospital charges are defined as the dollar amount a hospital charges for services prior to negotiating discounts with insurance companies. Hospital cost is the actual amount the hospital collects from the patient after the negotiated discounts. Secondary outcomes studied included rate of early endoscopy (defined as less than 1 day), AKI, blood transfusion, sepsis, ICU admission and TIPS (Transjugular Intrahepatic Portosystemic Shunt).

**Figure 1.**
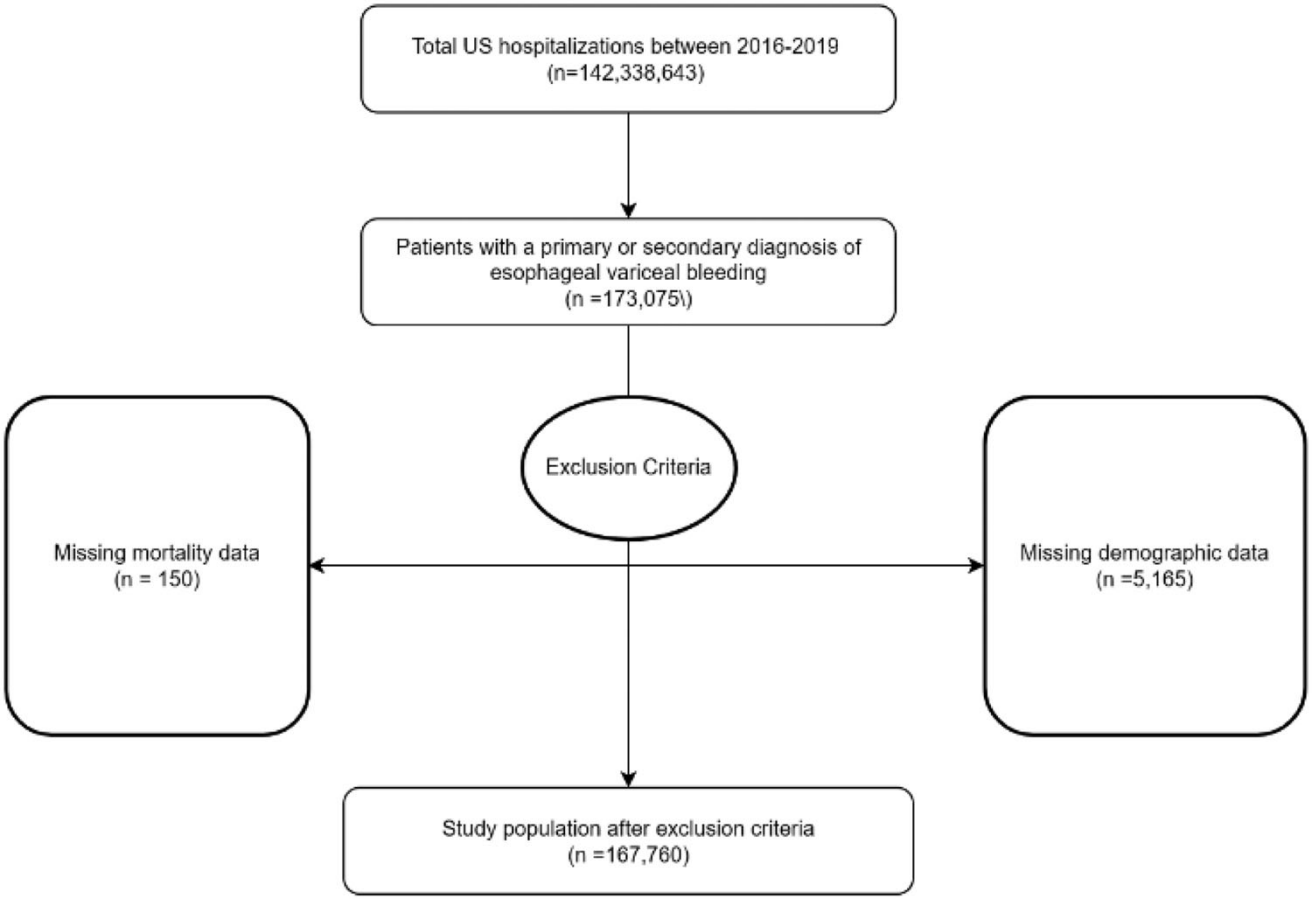
Flowchart of case selection for variceal bleeding, 2016–2019.

Our primary exposure variable was the gender of the patient. Other variables included age (divided into three groups; <44 years, 45–64 years and >65 years), race, primary insurance, median income, hospital characteristics such as region, bed size, rural/urban location pre-specified by HCUP, and transfer status. Data was also collected on the common causes of liver disease such as alcohol-related liver disease, hepatitis B, hepatitis C and alcohol-related hepatitis. To assess comorbidities, the Elixhauser Comorbidity Index was used [15]. This is a well-validated index based on ICD-10-CM codes meant to be used in large administrative data to predict mortality and hospital resource use. The index has 31 comorbid categories. We also studied the various decompensations of the disease including ascites, hepatic encephalopathy, spontaneous bacterial peritonitis, hepatorenal syndrome, and hepatocellular carcinoma. Finally, common etiologies leading to varices such as alcohol-related liver disease, hepatitis B, hepatitis C and alcohol-relatedc hepatitis were also studied. A complete list of ICD-10 codes is presented in the supplementary table (Figure 1).

### Statistical analysis

Hospital-level discharge weights provided by NIS were used to generate national estimates. Categorical variables were compared using the chi-square, whereas an independent sample t-test was used for continuous variables. Univariate logistic regression was done to study the effect of gender on categorical outcomes. It was followed by a multivariate logistic regression while adjusting for patient demographics, hospital characteristics, each of the elixhauser comorbidity, etiologies of the liver disease (as described in the variables), and liver decompensations. A complete list of Elixhauser comorbidities is presented in the supplementary table. Similarly, for continuous variables, univariate and multivariate linear regression was done. Similar to our logistic regression model, we adjusted for patient demographics, hospital characteristics, each of the Elixhauser comorbidities and liver decompensations. The unadjusted and odds ratio were calculated with a 95% confidence interval. A type I error of <0.05 was considered statistically significant. Data analysis was done using STATA 17.0 (Texas).

## Results

### Patient demographics

A total of 166,760 patients were included in the study. Females accounted for 32.7% of the study population. The mean age for women was 58.45, compared to 55.65 for men. The majority of the women admitted were 45 to 64 years old (53.03%). A higher proportion of females than males was present in the >65 age group. (31.95% vs 22.05%). The majority of the women admitted with variceal bleeding were White (67.1%) followed by Hispanic (17.91%). A complete list of patient demographics is presented in Table 1.

**Table 1.** Patient demographics and hospital characteristics stratified by gender.

Variables	Men (*n* (%))	Women (*n* (%))	All (*n* (%))	*p* Value
**Patient age (yr)**	55.65 (+/−0.09)	58.45 (+/−0.13)	56.57 (+/−0.08)	**<.001**
**Age categories**				**<.001**
Age <44	19,360 (17.26)	8,200 (15.02)	27,560 (16.53)	–
Age 45-64	68,055 (60.68)	28,955 (53.03)	97,010 (58.17)	–
Age >65	24,745 (22.06)	17,445 (31.95)	42,190 (25.3)	–
**Race**				**<.001**
White	68,890 (61.42)	36,635 (67.1)	10,5525 (63.28)	–
Black	8,375 (7.47)	3,640 (6.67)	12,015 (7.21)	–
Hispanic	25,660 (22.88)	9,780 (17.91)	35,440 (21.25)	–
Asian or Pacific Islander	2,775 (2.47)	1,365 (2.5)	4,140 (2.48)	–
Native American	2,245 (2)	1,550 (2.84)	3,795 (2.28)	–
Other	4,215 (3.76)	1,630 (2.99)	5,845 (3.51)	–
**Primary payer**				**<.001**
Medicare	34,210 (30.5)	21,430 (39.25)	55,640 (33.37)	–
Medicaid	33,510 (29.88)	14,570 (26.68)	48,080 (28.83)	–
Private and HMO	27,710 (24.71)	13,115 (24.02)	4,0825 (24.48)	–
Self Pay	11,315 (10.09)	3,720 (6.81)	15,035 (9.02)	–
**Hospital bed size**				.121
Small	18,960 (16.9)	9,255 (16.95)	28,215 (16.92)	–
Medium	33,555 (29.92)	15,745 (28.84)	49,300 (29.56)	–
Large	59,645 (53.18)	29,600 (54.21)	89,245 (53.52)	–
**Hospital teaching status**				.074
Non-Teaching	30,075 (26.81)	15,160 (27.77)	45,235 (27.13)	–
Teaching	82,085 (73.19)	39,440 (72.23)	121,525 (72.87)	–
**Hospital region**				**.001**
Northeast	16,460 (14.68)	7,480 (13.7)	23,940 (14.36)	–
Midwest	18,825 (16.78)	10,060 (18.42)	28,885 (17.32)	–
South	46,770 (41.7)	22,715 (41.6)	69,485 (41.67)	–
West	30,105 (26.84)	14,345 (26.27)	44,450 (26.66)	–
**Transfer status**				.292
Not transferred	95,055 (84.75)	45,875 (84.02)	140,930 (84.51)	–
From acute care	14,005 (12.49)	7,150 (13.1)	21,155 (12.69)	–
Other facility	2,600 (2.32)	1,365 (2.5)	3,965 (2.38)	–
**Rural/Urban location**				.682
Rural	106,630 (95.07)	51,850 (94.96)	158,480 (95.03)	–
Urban	5,530 (4.93)	2,750 (5.04)	8,280 (4.97)	–

### Etiology, comorbidities and complications of cirrhosis

Females had lower rates of coagulopathy, ascites and hepatocellular cancer than males. The incidence of alcohol-related liver disease, NASH, Hepatitis B, and Hepatitis C related liver disease was lower in females when compared to males. A complete list of liver etiologies and decompensations of liver disease is present in Table 2.

**Table 2.** Elixhauser comorbidities, liver etiologies and complications of cirrhosis stratified by gender.

Variables	All (*n* (%))	Male (*n* (%))	Female (*n* (%))	*p* Value
Elixhauser comorbidities				.003
1	1,750 (1.05)	1,030 (0.92)	720 (1.32)	–
2	9,935 (5.96)	6,730 (6)	3,205 (5.87)	–
≥ 3	155,075 (92.99)	104,400 (93.08)	50,675 (92.81)	–
**Liver etiology**				
Alcohol-related liver disease	91,300 (54.75)	69,375 (61.85)	21,925 (40.16)	**<.001**
NASH	5,140 (3.08)	3,105 (2.77)	2,035 (3.73)	**<.001**
Hepatitis B	3,725 (2.23)	2,995 (2.67)	730 (1.34)	**<.001**
Hepatitis C	35,555 (21.32)	26,245 (23.4)	9,310 (17.05)	**<.001**
Alcohol-related hepatitis	22,435 (13.45)	16,480 (14.69)	5,955 (10.91)	**<.001**
**Complications of cirrhosis**				
Ascites	74,360 (44.59)	50,490 (45.02)	23,870 (43.72)	**.0267**
SBP	5,515 (3.31)	3,815 (3.4)	1,700 (3.11)	.17
Hepatorenal syndrome	8,315 (4.99)	5,745 (5.12)	2,570 (4.71)	.10
Coagulopathy	89,720 (53.8)	61,685 (55)	28,035 (51.35)	**<.001**
Hepatocellular carcinoma (HCC)	8,160 (4.89)	6,825 (6.09)	1,335 (2.45)	**<.001**

## Outcomes

### All-Cause mortality

**Figure 2.**
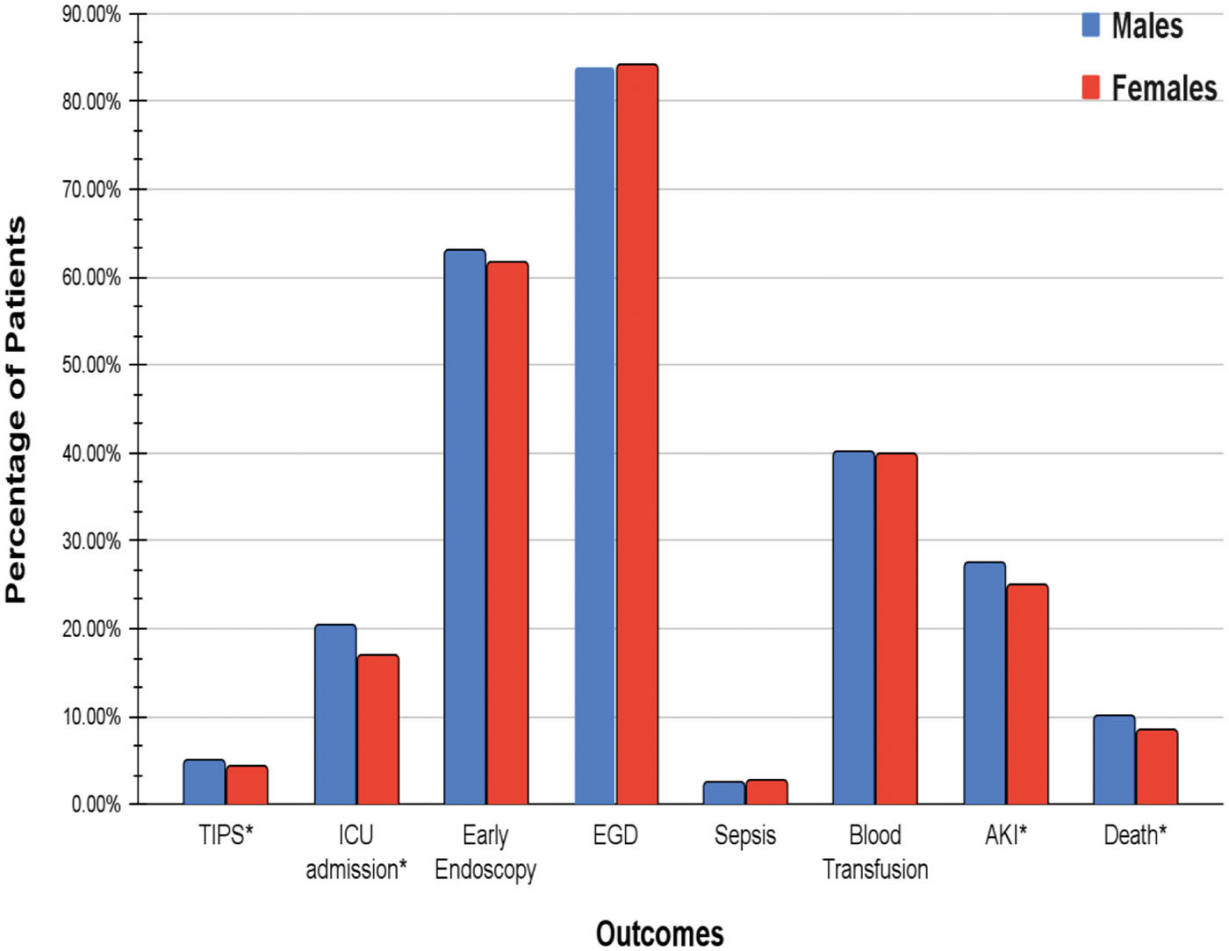
Categorical outcomes in patients admitted with variceal bleeding, stratified by gender (* signifies *p* < .05).

Total in-hospital mortality in the study population was 15,645 (9.38%). The mortality rate in males was 9.91% compared to 8.31% in females. There was a significant difference in mortality in patients based on gender (aOR = 0.88, *p* = .008). The results of all-cause mortality and other outcomes are presented in Figure 2.

### Length of stay

The length of stay in the study population was 6.12 days. In males, the length of stay was 6.09 (±0.05) days compared to 6.18 (±0.08) days in females. There was no statistically significant difference between the two groups (adjusted coefficient = 0.1, *p*=.252).

### Mean hospitalization charges and costs

In males, the mean charges and costs were $89,398.79 and $20,762.7 compared to $87,052.14 and $20,236.93 in females, respectively. There was no statistically significant difference in hospitalization costs and charges between the two groups (adjusted coefficient=$2,135.59, *p*=.247, and adjusted coefficient $607.16, *p*=.183, respectively).

### Acute kidney injury

A total of 26.65% of the study population developed kidney injury. Acute kidney injury was documented in 27.54% of males and 24.83% of females. On multivariate analysis, women had a statistically significant decrease in the odds of developing AKI compared to males (aOR = 0.82 *p*<.001).

### ICU admission

A total of 19.25% of the study population was admitted to the ICU. ICU admission was documented in 20.38% of the males and 16.93% of the females. Women had statistically significantly decreased odds of being admitted to the ICU compared to men (aOR = 0.84 *p*<.001).

### TIPS

A total of 4.65% of the study population required TIPS. TIPS was documented in 4.88% of males and 4.19% of females. Women had statistically significant lower odds of requiring TIPS than men (aOR = 0.83 *p*=.002).

### Endoscopy

A total of 83.94% of the study population underwent endoscopy. About 83.91% of the males and 84.01% of the females had endoscopic intervention performed during the hospitalization. No statistically significant difference was noted between the two groups (aOR = 0.99 *p*=.829).

A total of 62.5% of the study population had an endoscopy done within one day of hospitalization. Within one day of being hospitalized, 63% of males and 61.66% of females underwent an endoscopy. There was no statistically significant difference between the two groups in the odds of receiving early endoscopy (aOR = 0.98 *p*=.369).

### Sepsis

The total incidence of sepsis in the study population was 2.53%. About 2.48% of males and 2.65% of females developed sepsis. There was no statistically significant difference in the incidence of sepsis between the two groups (aOR = 1.13 *p*=.117).

### Blood transfusion

A total of 40.07% of the study population required blood transfusion. During hospitalization, 40.13% of the males and 39.95% of the females received blood products. There was no statistically significant difference noted in the odds of receiving blood products based on the gender of the patient (aOR = 1.03 *p*=.324). (Tables 3 and 4)

**Table 3.** Table showing unadjusted and adjusted odds ratio for study outcomes on univariate and multivariate analysis.

Variables	Unadjusted OR	*p* Value	Adjusted OR	*p* Value
Death	0.82	**<.001**	0.88	**.008**
EGD	1.01	.818	0.99	.829
Early EGD	0.94	**.02**	0.98	.369
Acute Kidney Injury	0.87	**<.001**	0.82	**<.001**
Blood transfusion	0.99	.76	1.03	.324
Sepsis	1.07	.36	1.13	.117
ICU admission	0.8	**<.001**	0.84	**<.001**
TIPS	0.85	**.005**	0.83	**.002**

**Table 4.** Table showing unadjusted and adjusted coefficients for continuous study outcomes on univariate and multivariate analysis.

Variable	Unadjusted coefficient	p-value	Adjusted coefficient	*p* Value
Length of Stay	0.1	.252	0.003	.975
Total Charge	2346.65	.211	2135.59	.247
Hospitalisation Costs	525.63	.259	607.16	.183
Time to Endoscopy	0.05	.199	0.02	.632

## Discussion

Esophageal variceal bleeding is associated with higher morbidity and mortality than other causes of gastrointestinal bleeding. Although mortality rates due to esophageal variceal bleeding are steadily declining, this complication continues to be one of the leading causes of death in patients with cirrhosis [10]. Previous studies investigating gender disparities in Italy and Norway have shown that women are at a lower risk of death when admitted with acute esophageal variceal bleeding [12,13]. To the best of our knowledge, our study is the first in the United States to evaluate gender differences in hospitalizations for esophageal variceal bleeding.

In our study population of 166,760 patients, 54,600 (32.7%) were female while the remaining were male (67.3%). Female gender was associated with a 12% decreased risk of in-hospital mortality after adjusting for confounding factors. Our results are consistent with the previously published international studies [12,13]. There are various possible explanations for these findings.

Previous studies have described that estrogen inhibits TGF-β and prevents liver fibrosis [16]. Due to the protective effect of estrogen, females may present with decompensated cirrhosis at a later age compared to males. These differences were also noted in our study, with the average age of females being higher than that of males. Female gender has also been found to be protective against the progression of liver fibrosis in premenopausal but not in postmenopausal women with hepatitis C [17]. Previous studies have also noted an increased risk of hepatitis B related reactivation and exacerbations in men compared to women [18]. Another possible reason for decreased progression of liver disease with hepatitis B and C could be a lack of additional aggravating factors in women such as HIV and excessive alcohol use [19]. Although the mechanism behind this gender difference is unclear, this may explain the increased incidence of hepatitis B and C related cirrhosis in men compared to women.

The incidence of alcohol-related liver disease was lower in women as compared to men. Multiple studies have documented that women are at higher risk of liver damage from alcohol secondary to decreased activity of alcohol dehydrogenase and lower volume of distribution of alcohol [20,21]. Interestingly, it has also been noted that women with alcohol-related liver disease are more abstinent than men, which might contribute to decreased progression of liver disease in women. A study on 393 patients with alcohol use revealed women to have higher abstinent rates and lower mortality compared to men [22]. Although there is no way to ascertain the proportion of patients who were abstinent, a similar effect might have contributed to decreased mortality in women.

Estrogen has also been shown to decrease portal pressures and has been proposed as a therapeutic agent for portal hypertension [23,24]. A study by Zhang *et al.* performed on rat models showed that treatment with estrogen improved the systemic and splanchnic hyperdynamic circulation due to alleviation of oxidative stress [24]. There is a probability that this decrease in portal pressure translates to better outcomes for females with esophageal varices.

Our study revealed no difference between the two groups in the rates of spontaneous bacterial peritonitis or hepatorenal syndrome, which are associated with high morbidity and mortality. However, an increased incidence of HCC and coagulopathy among men was seen, which might also be contributing to the increased mortality seen in male patients. Our findings of increased HCC in men are similar to previously published studies [25,26].

Our study also showed that males had more ICU admissions and TIPS than females further highlighting the disease severity in males. TIPS is associated with decreased in-hospital mortality in patients with variceal bleeding [27]. Despite the higher rates of TIPS in men, our study results did not demonstrate lower mortality rates in this group.

Previous studies have indicated that males are more prone to immune depression after trauma and hemorrhage [28]. Sepsis is a well-known complication of variceal bleeding. Haukeland *et al.* hypothesized that this immunosuppression in males may lead to higher rates of sepsis, which might lead to increased mortality in males with esophageal bleeding [12]. However, in our study, no statistically significant difference was noted between the incidence of sepsis among males and females.

Furthermore, Rattan *et al.* found an inverse relationship between telomere length, liver disease, and mortality. They found that men have shorter telomere lengths as compared to females. They also hypothesized that women have lower telomere attrition rates which could explain the lower mortality rates [29].

There might be a gender delay in seeking medical help for symptoms of gastrointestinal bleeding. Our study could not assess this due to its retrospective design. A recent study exploring time to presentation after the onset of symptoms of gastrointestinal bleeding in 3000 patients found no gender difference in proportions attending hospitals within six hours. However, more than a 48 hour delay (20% of the patients) was observed among men which could also contribute to the increased mortality seen in men [30]. Our study also reports no difference in the mean length of stay and total hospitalization cost between the two groups. Endoscopy and early endoscopy rates were also similar between the two groups

We acknowledge the following limitations of this study. First, NIS lacks objective data limiting our ability to calculate the severity of illness scores such as MELD-Na or Child-Pugh. To account for this limitation, decompensations from cirrhosis were used as surrogate markers of severity. This has been utilized and validated in prior studies [31–33]. Secondly, information on pharmacological therapies such as octreotide or prophylaxis with non-selective beta-blockers is not included in NIS, which are important confounders and may affect patient outcomes. In addition, NIS does not provide patient identifiers therefore we are unable to track readmissions. As a result, it is difficult to ascertain if the bleeding episodes were primary or rebleeding. Finally, data from NIS only includes acute hospitalization episodes therefore the patients cannot be followed longitudinally. The study's strength comes from the large population size and exclusion of sample bias from data collected from a single region or hospital. Our findings should be validated in a prospective cohort study that captures more granular clinical data including information on patterns of liver decompensations and long-term mortality.

Despite these limitations, our study expands on the current knowledge of gender disparities in patients with esophageal variceal bleeding. Our principle findings provide insight that men with esophageal variceal bleeding fare worse compared to women. This study reveals that prognosis depends not only on the etiology and complications of liver disease but also on gender. Further research is needed to elucidate the factors responsible for the reduced mortality in female patients. The development of gender-specific management programs would help target the needs of women and men living with cirrhosis.

## Supplementary Material

Supplemental Material

## Data Availability

The data supporting the findings of this study are available from the corresponding author upon reasonable request. This study was conducted in compliance with the ethical standards of the responsible institution on human subjects as well as with the Helsinki Declaration; or, this study was conducted in compliance with all the applicable institutional ethical guidelines for the care, welfare and use of animals.
