# Supplementary material for: Gender differences in esophageal variceal bleeding in the United States
Source: Ann Med. 2022 Aug 5;54(1):2115–22. doi: 10.1080/07853890.2022.2104920 (PMC9359179; doi:10.1080/07853890.2022.2104920)
Supplement: Supplemental Material [file IANN_A_2104920_SM8353.docx]

Supplementary Table

| **Variables** | **ICD-10 codes** |
| --- | --- |
| Bleeding Varices | I85.01, I85.11 |
| **Liver Etiology** |  |
| Alcohol-related liver disease | K70.0, K70.1 , K70.2, K70.4 |
| Hepatitis C | B17.1, B18.2, B19.2 |
| NASH | K75.81, K76.0 |
| Hepatitis B | B19.1, B16 , B18.1 |
| Alcoholic Hepatitis | K70.1, K70.4 |
| **Cirrhosis Complications** |  |
| Hepatocellular cancer | C22 |
| Spontaneous Bacterial Peritonitis | K65.2 |
| Ascites | R18, K71.51, K70.11, K70.31 |
| Hepatorenal syndrome | K76.7 |
| Coaguopathy | D65-D68.x, D69.1, D69.3-D69.6 |
| **Outcomes** |  |
| Endoscopy | 0D917ZX,0D917ZX,0D918ZX,0D927ZX,0D928ZX,0D937ZX,0D938ZX,0D947ZX,0D948ZX,0D957ZX,0D958ZX,0D967ZX,0D968ZX,0D977ZX,0D978ZX,0D987ZX,0D988ZX,0D997ZX,0D938ZX,0D998ZX,0D9A7ZX,0D9A8ZX,0D9B7ZX,0D9B8ZX,0D9C4ZX,0D9C7ZX,0D9C8ZX",0DB17ZX,0DB18ZX,0DB27ZX,0DB28ZX,0DB37ZX,0DB38ZX,0DB47ZX,0DB48ZX,0DB57ZX,0DB58ZX,0DB67ZX,0DB68ZX,0DB77ZX,0DB78ZX,0DB97ZX,0DB98ZX,0DD18ZX,0DD28ZX,0DD38ZX,0DD48ZX,0DD58ZX,0DD68ZX,0DD78ZX,0DD98ZX,0DDA8ZX,0DDB8ZX,0DDC8ZX,0DJ08ZZ,0DJ68ZZ,06L34CZ,06L38CZ |
| Acute Kidney Injury | N17.0, N17.1 , N17.2, N17.8, N17.9 |
| Pressor | 3E030XZ,3E033XZ,3E040XZ,3E043XZ,3E050XZ,3E053XZ,3E060XZ,3E063XZ |
| Shock | R65.21, R57.1, R57.8, R57.9 |
| Mechanical Ventilation | 5A1935Z, 5A1945Z, 5A1955Z |
| Sepsis | R65.10, R65.11, R65.20 |
| Blood Transfusion | 302** |
| TIPS | 06183J4, 06184J4,06183JY,06183DY |
| ICU | Pressor + Mechanical Ventilation |
